# Acoustic micro-vortexing of fluids, particles and cells in disposable microfluidic chips

**DOI:** 10.1007/s10544-016-0097-4

**Published:** 2016-07-21

**Authors:** Ida Iranmanesh, Mathias Ohlin, Harisha Ramachandraiah, Simon Ye, Aman Russom, Martin Wiklund

**Affiliations:** 1 Department of Applied Physics, Royal Institute of Technology, KTH-Albanova, SE-106 91 Stockholm, Sweden; 2 School of Biotechnology, Royal Institute of Technology, KTH-SciLifeLab, SE-171 21 Solna, Sweden

**Keywords:** Acoustic streaming, Lab on a chip, Cell lysis

## Abstract

We demonstrate an acoustic platform for micro-vortexing in disposable polymer microfluidic chips with small-volume (20 μl) reaction chambers. The described method is demonstrated for a variety of standard vortexing functions, including mixing of fluids, re-suspension of a pellet of magnetic beads collected by a magnet placed on the chip, and lysis of cells for DNA extraction. The device is based on a modified Langevin-type ultrasonic transducer with an exponential horn for efficient coupling into the microfluidic chip, which is actuated by a low-cost fixed-frequency electronic driver board. The transducer is optimized by numerical modelling, and different demonstrated vortexing functions are realized by actuating the transducer for varying times; from fractions of a second for fluid mixing, to half a minute for cell lysis and DNA extraction. The platform can be operated during 1 min below physiological temperatures with the help of a PC fan, a Peltier element and an aluminum heat sink acting as the chip holder. As a proof of principle for sample preparation applications, we demonstrate on-chip cell lysis and DNA extraction within 25 s. The method is of interest for automating and chip-integrating sample preparation procedures in various biological assays.

## Introduction

Vortexing is a standard method for manual sample preparation in biotechnology research and clinical laboratories, e.g. when re-suspending a pellet of centrifuged micro-beads suspended in a test tube. Technology for miniaturizing and automating such procedures are referred to as Lab on a Chip, often based on microfluidic chips. However, vortexing in microfluidics is more difficult to implement due to the small dimensions and strong viscous forces. While most studies in microfluidics have focused on mixing of fluids by various active or passive techniques (Lee et al. 2011), there are few simple and generic methods available for micro-vortexing in lab-on-a-chip devices. Such a method would be attractive since, in addition to simple fluid stirring, standard vortexing also involves stronger mechanical forces capable of, e.g., disintegrating aggregated micro-beads and disruption of cell membranes.

Acoustic methods have been used previously for fluid mixing in microfluidic systems (Yang et al. 2001; Wiklund et al. 2012). Examples include mixing by integrated transducers (Yaralioglu et al. 2004), oscillating sidewall sharp-edges (Huang et al. 2013) and oscillating trapped bubbles (Ahmed et al. 2009). These methods are impressive and efficient, but require purpose-made microchips or integrated transducers with specific geometrical features, which limit their applicability. Furthermore, cell lysis has previously been performed in miniature chambers by various techniques (Nan et al. 2014) including acoustic methods, e.g., lysis of bacterial spores by either sonication at 40 kHz in a cartridge combined with a filter (Taylor et al. 2001), or by sonication at 1.4 MHz with a dual-transducer system submerged in a water tank (Warner et al. 2009). However, both these systems have rather complex physical structure that makes them difficult to integrate in microfluidics. Fully chip-integrated acoustic methods for cell lysis have been demonstrated by, e.g., bulk acoustic waves in a 2.5-μl-channel microfluidic device operated at high-frequency (380 MHz) for lysis of cells and bacterial spores (Marentis et al. 2005), and by 9.5-MHz surface acoustic waves in a device combined with a phononic lattice used to produce a rotational-symmetry acoustic wave coupled into an oil-covered 3-μl droplet containing the cell sample (Reboud et al. 2012). These methods are sophisticated but require advanced chip-integrated transducers and specific chip designs.

In this paper, we demonstrate an ultrasound-based multi-purpose micro-vortexing platform designed for sample preparation in disposable polymer microfluidic chips with simple channel geometries. The platform consists of a modified temperature-regulated Langevin-type ultrasonic horn transducer, which is brought into contact with a microfluidic chip containing a 20 μl reaction chamber, and driven by a low-cost electronic board operating at fixed voltage and frequency. When the transducer is actuated, high-velocity (~cm/s) acoustic streaming is generated in the chamber, which is used for various on-chip micro-vortexing applications such as mixing of laminar flows, resuspending a pellet of magnetic beads, and cell lysis for DNA extraction.

## Transducer design, modelling and characterization

The actuation system originated from a ~ 50-Euro transducer – driver board system (UT-100, NK, Zhejiang, China) operating at fixed frequency 28 kHz. The original transducer consisted of two sandwiched ring-shaped piezoceramic plates (outer and inner diameters 45 and 15 mm, respectively), each with thickness 5.0 mm, clamped between a backside steel block and a frontside aluminum block.

### Transducer modelling

**Fig. 1:**
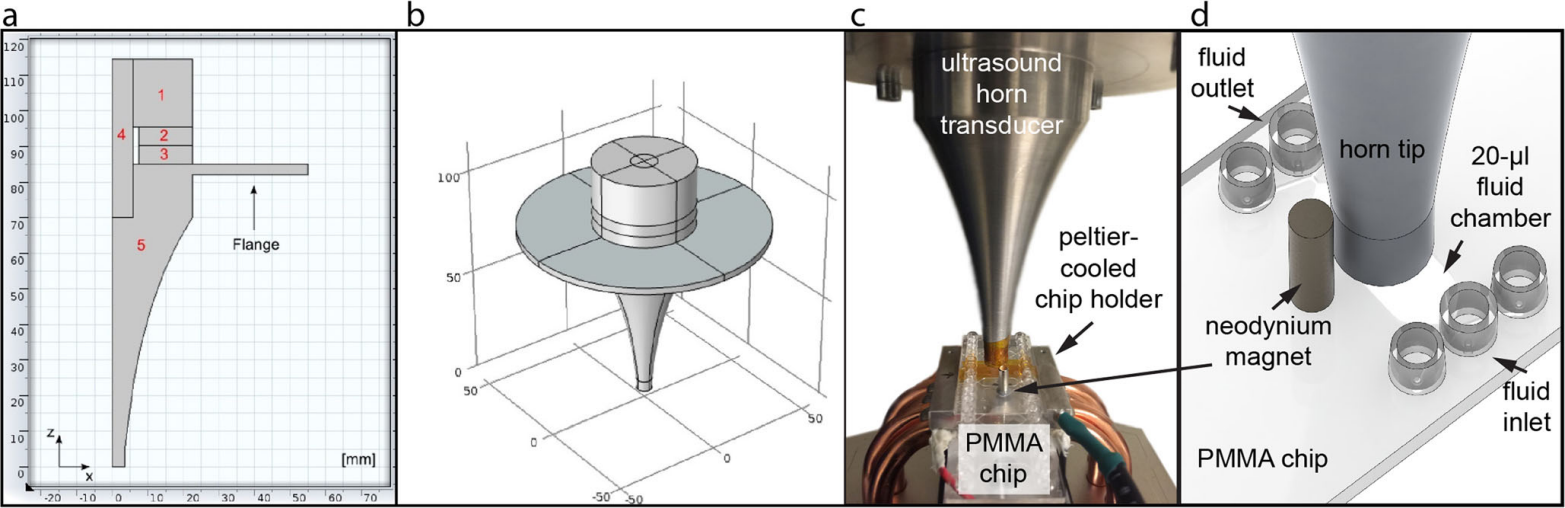
**a** The geometries and materials of the ultrasonic transducer parts used in the numerical modelling. (1): Stainless steel reflector (AISI 4340), (2) and (3): Upper and lower piezoelectric ceramic ring (Pz26, Ferroperm Piezoceramics A/S, Denmark), (4): Stainless steel compression bolt (AISI 4340), (5): Aluminum exponential horn with flange (6063-T83). Half the cross-section of the transducer (**a**), and illustration the full 3D model (**b**). Photo (**c**) and illustration (**d**) of the device for acoustic micro-vortexing in disposable PMMA chips. The photo shows the whole setup, while the illustration shows the PMMA chip placed in the chip holder and the lower part of the exponential tapered horn. The yellow color on the horn tip and the chip is a tape used for thermal imaging camera temperature monitoring

The transducer modification consisted of replacing its frontside block with an aluminum horn having a tip matching the dimensions of the microfluidic chip, see Fig. 1. For the transducer design we used finite element analysis with the software package COMSOL (version 4.3b, build 4.3.2.189). The transducer is simulated in an isolated environment, in order to find a design which has a resonance frequency matching that of the driver board, and also to find a design which generates a large displacement amplitude at the surface in contact with the chip. For this purpose, we selected a rotational-symmetric exponential-tapered design of the frontside block (the horn) including a flange for mounting the transducer in a positioning stage (see Fig. 1a and b). The Physics module *Acoustic-Piezoelectric Interaction, Frequency Domain* (acpz) was used for modelling the electrical impedance of the transducer. We used the following boundary conditions (with domains referring to Fig. 1a): Electrical → Ground (between domains 1 and 2); Electrical → Terminal (1 V_rms_) (between domains 2 and 3); Electrical → Zero Charge (exterior surfaces of domains 2 and 3); Electrical → Ground (between domains 3 and 5); Structural → Free (remaining surfaces). The default tetrahedral element was used for meshing the whole model with typically 5–10 elements per wavelength in each material, in order for the FEM solution to converge. We studied the frequency response in the range 10–100 kHz, covering the fundamental frequency (which should be close to the driving frequency at 28 kHz) and a few higher harmonics.

### Modelling the resonance frequency of the transducer

When optimizing the geometry of the different domains in Fig. 1a, we used a fixed shape and size of all domains except for domain 5 (the exponential horn) which was varied until we reached a modelled resonance frequency (*f*_mod_ = 28.15 kHz) close to the fixed driving frequency of 28 kHz. For this choice of geometries, the full assembled transducer has a total length corresponding to a half wavelength at the selected driving frequency. For this design, the maximum vibrations are located at the upper and lower surfaces of the full transducer, while there is a displacement node close to the piezoelectric elements and the flange. This design reduces the risk of damaging the piezoelectric elements by vibration, and the risk of coupling vibrations into the transducer positioning stage attached to the flange.

### Modelling the displacement of the transducer

**Fig. 2:**
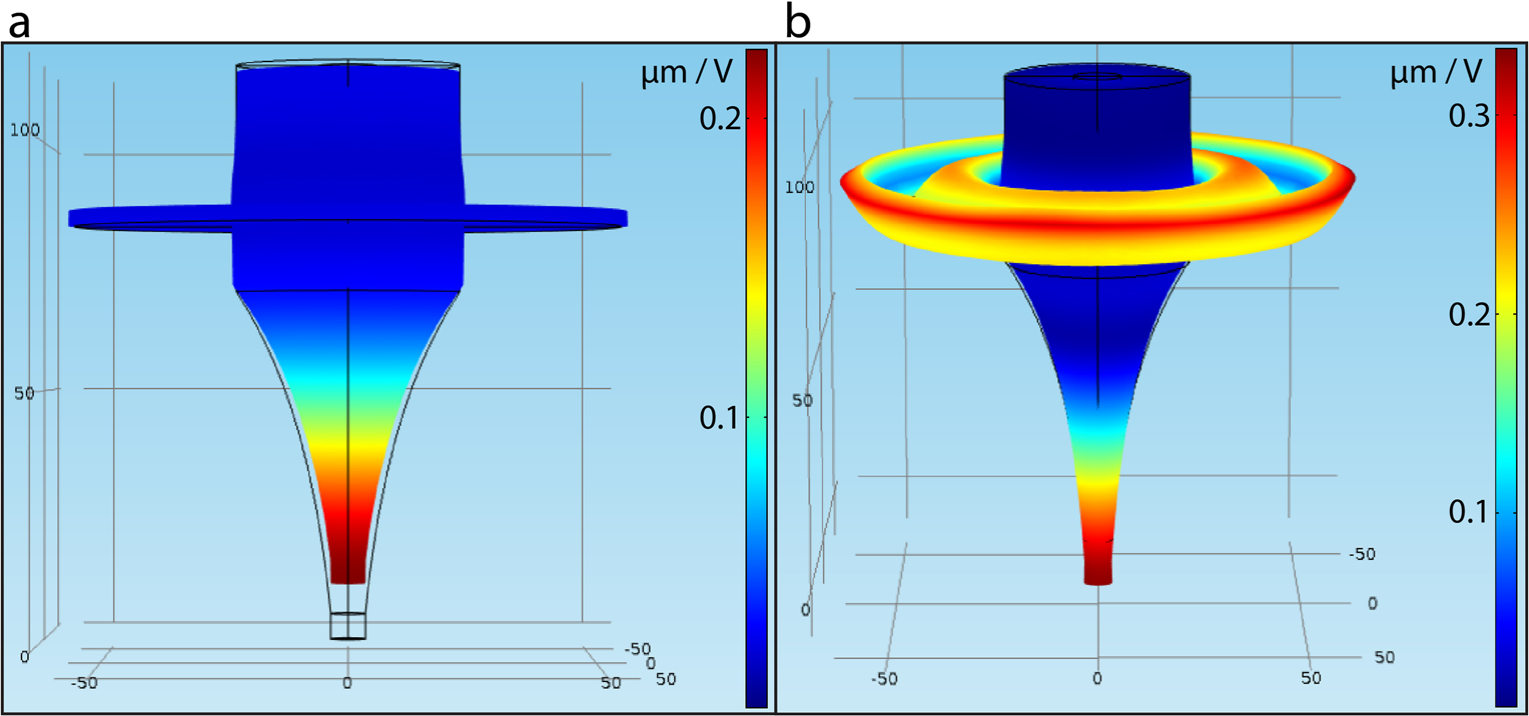
Modelled total displacement of the transducer per 1 V_rms_ actuation voltage at the fundamental resonance frequency, *f*_mod_ = 28.150 kHz (**a**), and at the first harmonics, *f*_mod_ = 37.425 kHz (**b**)

Figure 2 shows the total displacement of the transducer at the fundamental resonance frequency (*f*_mod_ = 28.15 kHz), and at the first harmonics (*f*_mod_ = 37.43 kHz), when it is driven by an input voltage of 1 V_rms_. As seen in Fig. 2a, the transducer operates in a pure longitudinal mode at the fundamental resonance frequency. We also note that the maximum displacement is located at the tip of the horn, while there is a minimum displacement in the upper region including the flange, piezoelectric elements and the reflector. The total displacement at the tip of the transducer is of the order of 10^−7^ m/V. Similar displacement amplitude at the tip is given at the first harmonics (Fig. 2b), but here we obtain another maximum displacement located in the flange including a lateral vibration mode. The modelled displacement is given for the actuation voltage 1 V_rms_, while the measured actuation voltage of the fixed power and frequency driving board was 1520 V, as measured with a high-voltage probe (Tektronix P6015A). This means that if we assume a linear relationship between the driving voltage and displacement, and optimal impedance matching between the driver board and the transducer, we may expect a displacement in our experiments of the order of 0.1 mm (maximum 0.3 mm). It is worth noting that this value has the same order of magnitude as the selected glycerol-filled distance between the transducer and the chip (cf. Sect. 3), which resulted in a robust and repeatable ultrasonic actuation.

### Electrical admittance measurements

An impedance analyzer (Z-Check 16,777 k, SinePhase, Austria) was used to experimentally measure the conductance versus frequency of the transducer and compare with the numerical simulations. The transducer was assembled by applying a torque of 70 Nm to the bolt (cf. domain 4, Fig. 1a), which resulted in a good acoustic coupling between the domains without the need for any glue. At this bolt compression, we measured an experimental resonance frequency of *f*_exp_ = 28.10 kHz, which was close to the modelled value (*f*_mod_ = 28.15 kHz).

## Experimental arrangement

The sample preparation platform – including the modified transducer, the chip and a cooling system – is shown in Fig. 1c and d. A small droplet (50 μl) of glycerol was placed between the horn and the chip for improving the acoustic coupling into the fluid chamber, and a glycerol-filled distance of 0.1 mm between the horn and the chip was kept to maintain a robust performance during transducer operation. The transducer was mounted in a holder with an *x-y-z* precision positioning stage, and was excited by the electronic driver board. In all experiments we used disposable PMMA microfluidic chips from Microfluidic ChipShop GmbH (Jena, Germany) containing a 20 μl reaction chamber and fluid inlet and outlet channels. The temperature on the transducer horn was monitored with a thermal imaging camera (FLIR C2, FLIR Systems, Inc., USA) and the temperature on the chip was monitored with a T-type micro-thermocouple (IT-21, Physitemp Instruments, USA) connected to a data logging unit (P655-LOG, Dostmann Electronic GmbH, Germany). For the cell lysis experiments, the chip was placed in a temperature-controlled holder, consisting of a Peltier regulator (TC0806, Cooltronic GmbH, Switzerland), a Peltier element (QC-127-1.4-6.0MS, Quick-Ohm Küpper & Co. GmbH, Germany) and a PC fan-cooled aluminum heat sink acting as the chip holder (cf. Fig. 1a). Before turning on the transducer, the temperature on the aluminum holder was adjusted to approx. 0 °C and monitored by a miniature PT1000 resistive temperature sensor (00409849, Jumo GmbG & CO. KG, Germany) attached with a thermal adhesive (Artic Silver Thermal Adhesive, Artic Silver Inc., USA). For the fluid mixing and magnetic bead vortexing experiments, no active cooling was used, which enabled imaging of the fluid chamber with an inverted microscope (Axiovert 40 CFL, Zeiss, Germany) equipped with a CCD camera (Sony α-7) and 1 × objective.

## Experimental results and discussion

### Temperature dependence of the acoustic actuation time

**Fig. 3:**
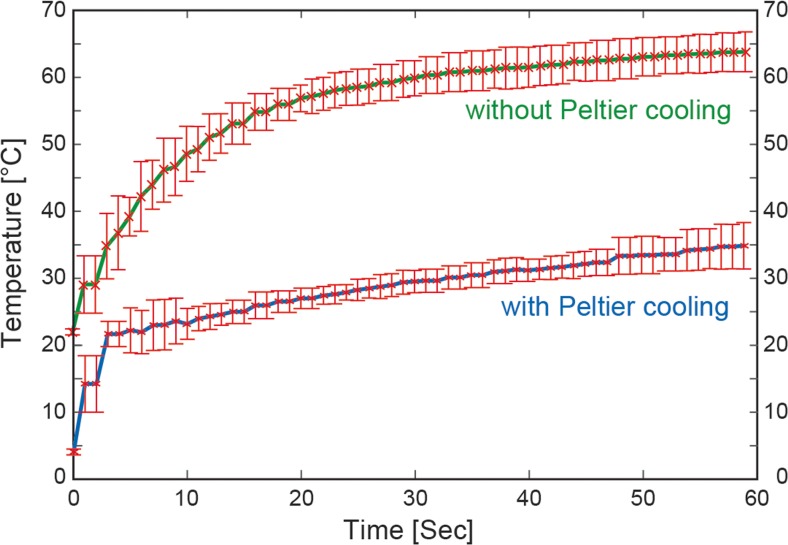
Temperature measured with a micro-thermocouple attached to the chip close to the fluid chamber as a function of transducer actuation time without (*green*) and with (*blue*) Peltier cooling turned on (*n* = 5)

The temperature was measured with and without active Peltier cooling of the chip by the use of the micro-thermocouple, see Fig. 3. As seen in the diagram (based on five repetitions of each experiment), without the active Peltier cooling the temperature increases from room temperature (22.0 ± 0.5 °C) when the transducer is turned on, up to 59.9 ± 2.6 °C after 30 s of actuation and 63.8 ± 3.0 °C after 1 min of actuation. When using the Peltier cooling system, the corresponding temperatures on the chip were 4.1 ± 0.4 °C (start), 29.6 ± 1.7 °C (30 s actuation) and 35.0 ± 3.5 °C (1 min actuation). This means that it is possible to operate the device for 1 min without exceeding physiological temperatures (37 ± 1 °C) (Wiklund 2012).

**Fig. 4:**
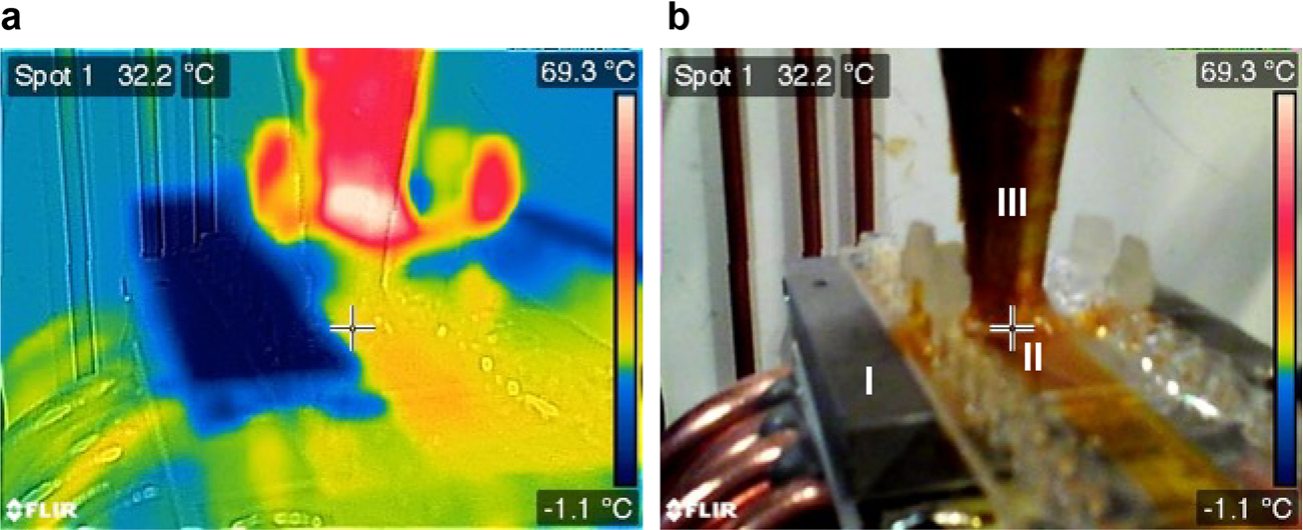
Thermal infrared image (**a**) and real visible-light image (**b**) of the system after 1 min of ultrasonic actuation. The system includes (*I*): The Peltier-cooled aluminum heat sink acting as the chip holder, (*II*): The chip, and (*III*): The exponential horn of the Langevin-type ultrasonic transducer. The orange part of the chip in (**b**) is a tape used for the thermal imaging camera (cf. Fig. 1a)

As a complement to the pointwise temperature measurements with the micro-thermocouple (cf. Fig. 3), we also measured the final temperature distribution of the Peltier-cooled system after 1 min of ultrasonic actuation by the use of the thermal imaging camera. The camera displays the minimum, maximum and center temperatures, see Fig. 4a. By pointing the central spot of the image (marked with a cross in Fig. 4) on the lowest part of the horn transducer in contact with the chip, we get an estimate of the maximum temperature on the chip. Furthermore, as seen in Fig. 4a, the minimum temperature in the image corresponds to the visible upper part of the aluminum heat sink attached on top of the Peltier element (Fig. 4, ‘I’), while the maximum temperature in the image corresponds to the central part of the exponential horn of the Langevin-type ultrasonic transducer (Fig. 4, ‘III’). Based on five repetitions of the experiment exemplified in Fig. 4, we conclude that although there is a relatively large temperature gradient between the aluminum heat sink and the transducer, the chip temperature measured with the thermal imaging camera (32.1 ± 3.6 °C) is in good agreement with the chip temperature measured with the thermocouple probe (35.0 ± 3.5 °C).

### Mixing of two laminar flow fluids

**Fig. 5:**
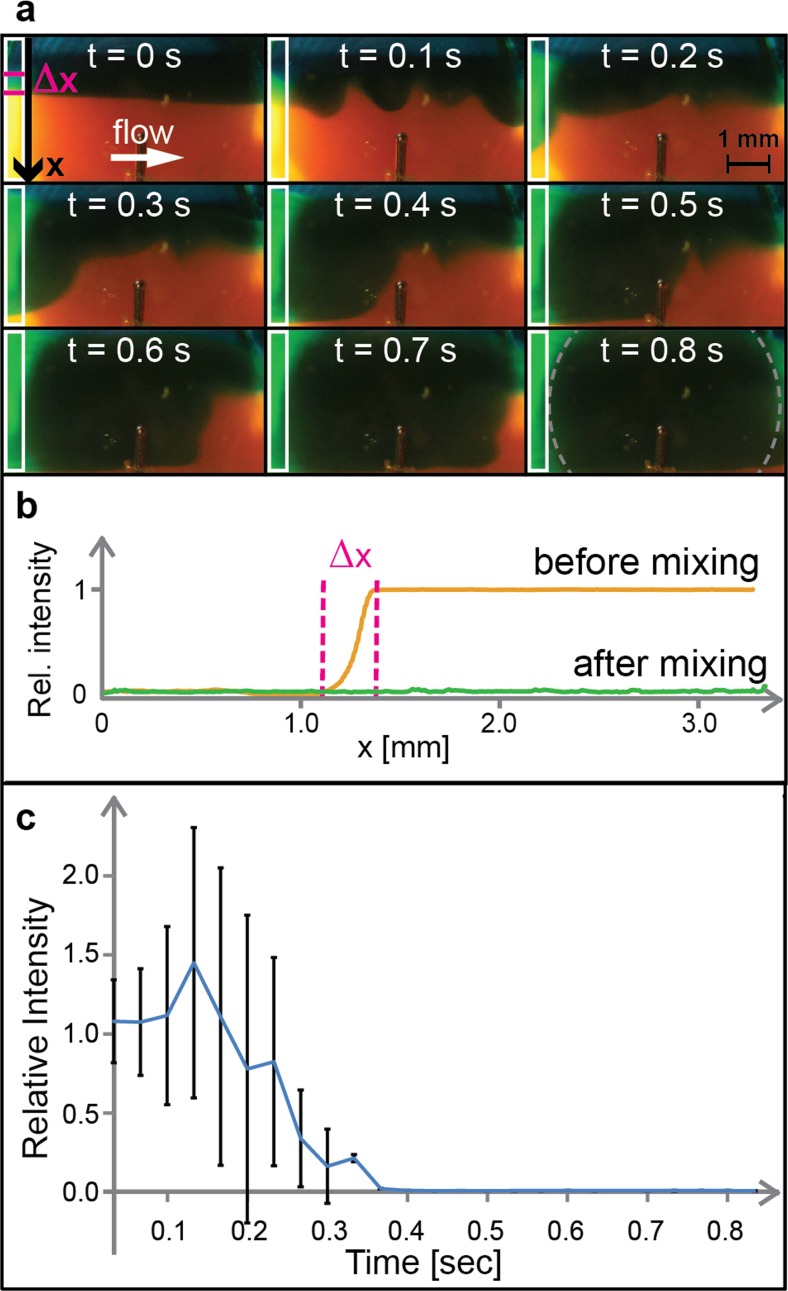
Acoustic mixing of two parallel laminar flows in the reaction chamber. **a** Views from the reaction chamber during the mixing process. Here, the gray dotted lines in the last image mark the transducer tip position which causes a shadow of the illumination light; for this reason we analyzed the parts outside the region marked with white boxes. **b** Relative light intensity in the red channel from the first and last RGB-images in (**a**) as a function of the lateral position x across the chamber taken before the transducer was turned on. Here, Δx is the pre-mixed region due to diffusion. **c** Relative light intensity in the red channel averaged along x as a function of transducer actuation time from four repetitions of the experiment, revealing an average mixing time in the analyzed region (*white boxes*) of less than 0.4 s. The *blue curve* is the average intensity (*n* = 4), and the values are normalized to the first repetition

In the first set of vortexing experiments, we demonstrate how to use the device for mixing of two parallel laminar flows driven through the chamber at 200 μl/min by a syringe pump. The fluids consisted of water with two different food coloring (blue and yellow), resulting in green color when mixed. As seen in Fig. 5, the two fluids were mixed on average within less than 0.4  s after the transducer was turned on (four repetitions of the experiment). For quantification of the mixing we analyzed the average pixel value across the width of the chamber in the red channel of the RGB-image frames from recorded video clips. As seen in the diagram in Fig. 5b, the red channel had a high pixel value in the yellow fluid but low value in both the blue and in the resulting mixed green fluid. The quantification in Fig. 5c is valid within the analyzed region marked with white boxes in Fig. 5a. At the selected flow rate (200 μl/min), the full mixing time in the whole 20-μl chamber was approx. 0.8 s (cf. Fig. 3a). However, at this flow rate it takes at least 6 s (>> 0.8 s) to exchange the full volume in the chamber. Thus, we may conclude that the mixing takes place in the whole reaction chamber and not only within the analyzed region marked with white boxes in Fig. 5a. When analyzing the velocity of color change in different regions in the chamber, we conclude that the acoustic vortexing causes rotational fluid velocities up to approx. 10 mm/s. By analyzing the measurements in Fig. 3, we note that the temperature increase during the first second of ultrasonic actuation is 7.1 ± 4.5 °C. Thus, for actuation times lasting for fractions of a second, a temperature increase of a few degrees is expected. However, for longer actuation times, active cooling is needed if the sample mixing is used in a temperature-sensitive assay.

### Vortexing of magnetic beads

**Fig. 6:**
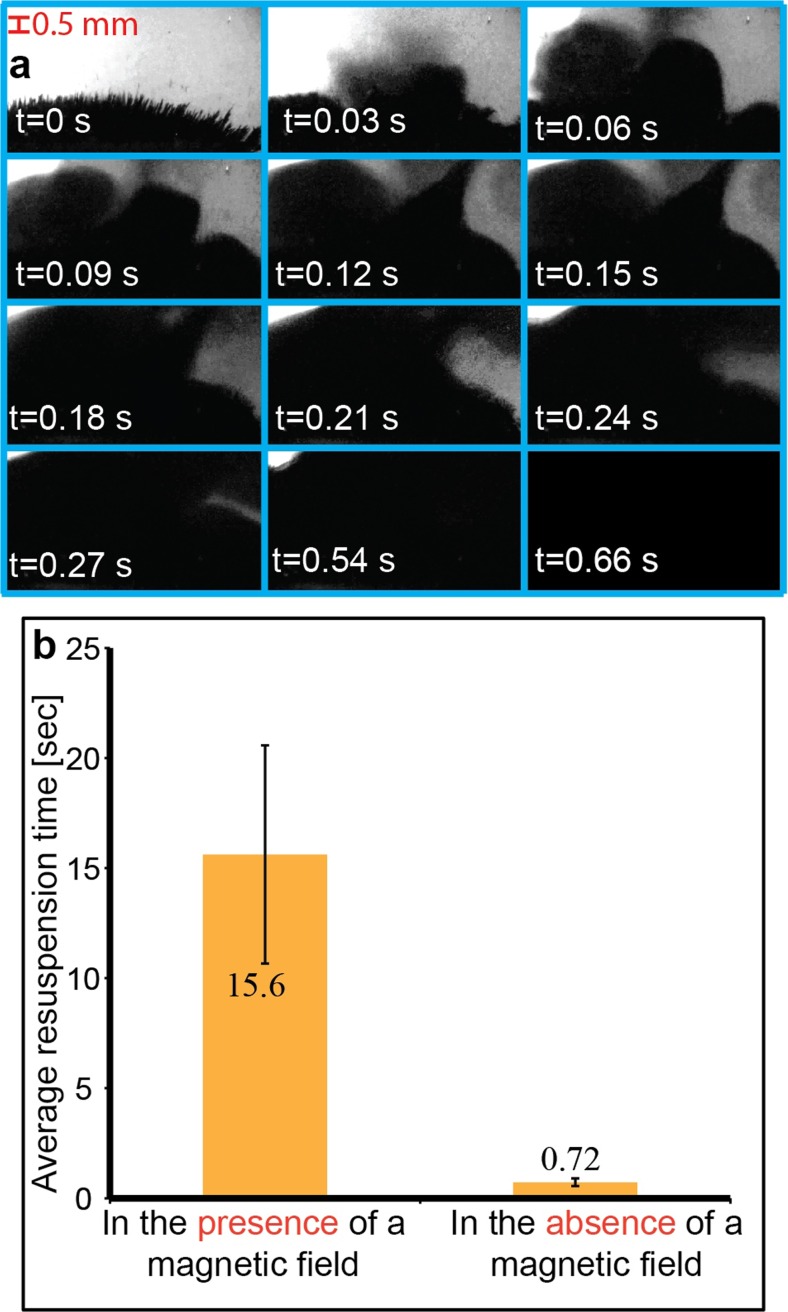
**a** Resuspension of a pellet of magnetic beads by acoustic micro-vortexing inside the chamber. The 300-nm beads are initially clumped at one of the sides of the chamber by the magnet. At *t* = 0 s, the magnet has been removed and the transducer is turned on. Complete resuspension occurs within 0.66 s in this experiment. **b** Quantification (*n* = 4) of the resuspension time of the magnetic bead pellet in the presence of a magnetic field (magnet remaining on the chip), and in the absence of a magnetic field (magnet removed from the chip, same conditions as in (**a**))

In the next set of experiments, we demonstrate one of the most common manual vortexing functions: To disaggregate and re-suspend a pellet of packed beads back into a homogeneous particle suspension. This standard vortexing function is traditionally performed for washing purposes in various bead- or cell-based assays by manually pushing, e.g. an Eppendorf tube onto a quickly oscillating disc to produce a fast rotating flow inside the tube. Here, we first trapped 300 nm-sized magnetic beads (MagSi-DNA-beads, MagnaMedics Diagnostics B.V., Geleen, The Netherlands) diluted to 3 mg/ml in Dulbecco’s phosphate-buffered saline (DPBS) into a pellet at one of the side walls of the reaction chamber in the chip by a rod-shaped Neodynium magnet (diam. 3 mm, height 8 mm) placed as close as possible to chamber (cf. Fig. 1d). The experiment was performed with the magnet removed (cf. example in Fig. 6a) and with the magnet remaining on the chip. The quantification was performed with the same method as for the fluid mixing in Fig. 5. As seen in Fig. 6b, the resuspension time was on average 0.7 s (vortexing time in the absence of a magnetic field) and 16 s (vortexing time in the presence of a magnetic field). When the magnet was removed, we note that the pellet resuspension time (Fig. 6) corresponds well with the full-chamber fluid mixing time (Fig. 5), both completed within less than a second. However, we note that acoustic vortexing of the pellet is also possible without the need for removing the magnet, which enables a simple procedure of on-chip bead washing with a fixed assembled setup (chip, magnet and ultrasound transducer).

### Cell lysis and DNA extraction

In the last set of experiments, we investigate whether our acoustic micro-vortexing method can be used for cell lysis as well as integrated DNA extraction. Here, we used the human lung cancer cell line A549 (adenocarcinoma human alveolar basal epithelial cells) suspended in DPBS buffer. The transducer was operated for 25 s per experiment. This actuation time was selected from a series of experiments investigating cell membrane integrity for different actuation times between 0 and 30 s. Here, cell lysis was confirmed visually, i.e. no viable cell was observed by bright-field microscopy in the chamber after 25 s. Hence, we conclude that up to 25 s is required to completely lyse mammalian cells in the device. As seen in Fig. 3, the temperature increase on the chip during 25 s without active cooling is on average 36 °C (from 22.0 ± 0.5 °C to 58.5 ± 2.7 °C). This temperature is within the normal working range in standard DNA extraction protocols. However, to exclude any lysis effects trigged by heating from the acoustic effects, we chose to operate the device with the chip placed in the Peltier-cooled chip holder (cf. Fig. 1c). As seen in Fig. 3, we note that the temperature on the chip with Peltier cooling did not exceed 30 °C during 25 s of acoustic actuation. After the acoustic cell lysis, we explored the possibility for extracting DNA by including magnetic beads as well as binding buffer. Following the lysis and DNA binding to the magnetic beads, an elution buffer was added to release the DNA for off-chip quantification. We compared an off-chip chemical lysis standard protocol (MagSi-DNA kit) with our on-chip acoustic lysis method. Based on four repetitions of the experiment, the amount of extracted DNA was 94.3 ± 6.7 ng/ml and 43.0 ± 7.8 ng/ml for the chemical and acoustic lysis methods, respectively. This corresponds to an average yield of 46 % of the acoustic method relative the chemical method. Although more experiments are needed to optimize the DNA binding buffer conditions, we demonstrate that DNA can be extracted within 25 s, and we show the possibility of integrating and simplifying a number of sample preparation steps using on-chip acoustic micro-vortexing.

## Conclusion

In conclusion, we have demonstrated a simple, generic and inexpensive method for micro-vortexing in micro-scale volumes by ultrasonic actuation of disposable polymer chips. In contrast to previous reports, the method does not require integration of transducers into the chip, nor does it require complex geometrical features of the channel or chamber in the chip. The device is based on a modified Langevin-type ultrasonic transducer and low-cost fixed-frequency electronic driver board capable of vortexing the full volume (20 μl) of the reaction chamber within less than a second. The major advantage with this actuation system is the several orders of magnitude lower cost in comparison to advanced tunable-frequency RF amplifiers often used in microscale acoustofluidic applications (Ohlin et al. 2013). From Comsol modelling, the estimated vibration amplitude at the transducer surface in contact with the chip is of the order of 0.1 mm (maximum 0.3 mm). It was not possible to determine whether this ultrasonic actuation caused cavitation in the reaction chamber. We did not observe any bubble activity within the time frames of the video recordings, we could only observe the fluid motion. The spatial and temporal resolution available with the microscope and camera used was not enough to monitor any potential μm-sized bubbles oscillating at 28 kHz. We also tried to use the sonoluminescent chemical Luminol as a cavitation probe (Johansson et al. 2016), but we did not observe any light signal from this probe (probably due to the too small volume of the reaction chamber). On the other hand, the transducer used in this work is intended for cavitation-based applications, primarily for ultrasonic cleaning, but in much larger volumes. Although it is difficult to predict the consequences when scaling down the actuated volume several orders of magnitude, it is highly likely that cavitation is also present in our micro-device. As seen in Fig. 3, the powerful method also causes a relatively quick temperature increase in the chip, which can be efficiently limited by a simple PC-fan, a Peltier regulator and an aluminum heat sink functioning as the chip holder. Although this is not needed for DNA extraction, our experiments demonstrate that acoustic micro-vortexing can be operated for 1 min below physiological temperatures. By selecting different actuation times, we demonstrated mixing of fluids (within less than a second), resuspension of a pellet of magnetic beads (within approx. 1–16 s, depending on if the magnet is removed or not), and lysis of cells as well as DNA extraction (within less 30 s). The proposed micro-vortexing method has the possibility to integrate a number of important and commonly used sample preparation steps for different lab-on-a-chip applications, including molecular diagnostics. In the future, we plan to combine the sample preparation method with polymerase chain reaction (Bartlett and Stirling 2003) (PCR)- and/or isothermal rolling circle amplification (Nilsson et al. 1994) (RCA)-based DNA analysis.
